# Navigating the science policy interface: a co-created mind-map to support early career research contributions to policy-relevant evidence

**DOI:** 10.1186/s13750-024-00334-5

**Published:** 2024-05-24

**Authors:** Carla-Leanne Washbourne, Ranjini Murali, Nada Saidi, Sophie Peter, Paola Fontanella Pisa, Thuan Sarzynski, Hyeonju Ryu, Anna Filyushkina, Carole Sylvie Campagne, Andrew N. Kadykalo, Giovanni Ávila-Flores, Taha Amiar

**Affiliations:** 1 Department of Science, Technology, Engineering and Public Policy, University College London, Gower Street, London, WC1E 6BT UK; 2 Geography Department, Humboldt-Universität zu Berlin, Unter den Linden 6, 10099 Berlin, Germany; 3 Snow Leopard Trust, 464, Sunnyside Ave N, Suite 325, Seattle, WA 998103 USA; 4 UNESCO Centre for Water Law, Policy & Science, University of Dundee, Nethergate, Dundee, DD1 4HN UK; 5 ISOE–Institute for Social-Ecological Research, Hamburger Allee 45, 60486 Frankfurt am Main, Germany; 6 Global Mountain Safeguard Research (GLOMOS), Institute for Environment and Human Security, United Nations University, Bolzano, Italy; 7 Centre for Global Mountain Safeguard Research (GLOMOS), Eurac Research, 1 Viale Druso, 39100 Bolzano, Italy; 8 Graduate School of Environmental Studies, Tohoku University, Sendai, Japan; 9 CIRAD (Centre de Coopération Internationale en Recherche Agronomique Pour le Développement), UMR DIADE, 34398 Montpellier, France; 10 BNZ Partners, 10, Gukhoe-daero 76-gil, Yeongdeungpo-gu, Seoul, Republic of Korea; 11 Institute for Environmental Studies (IVM), Vrije Universiteit Amsterdam, De Boelelaan 1111, 1081 HV Amsterdam, The Netherlands; 12 Sorbonne Université, CNRS, Station Biologique de Roscoff, UMR7144, Adaptation et Diversité en Milieu Marin, Sorbonne Université, Place Georges Teissier, 29680 Roscoff, France; 13 Institute of Physical Geography and Landscape Ecology, Leibniz Universität Hannover, Hannover, Germany; 14 Department of Biology and Institute of Environmental and Interdisciplinary Sciences, Carleton University, 15 1125 Colonel By Drive, Ottawa, ON K1S 5B6 Canada; 15 Department of Natural Resource Sciences, McGill University, Macdonald-Stewart Building, McGill, 21111 Lakeshore Road, Montreal, QC H9X 3V9 Canada; 16 Departamento Académico de Ciencias Marinas y Costeras, Universidad Autónoma de Baja California Sur, La Paz, Baja California Sur México; 17 University Felix Houphouet-Boigny, WABES Project, BP V34, 01, Abidjan, Côte d’Ivoire

**Keywords:** Biodiversity, Boundary organizations, Capacity building, Ecosystem services, Science-policy interface, Implementation science, Evidence

## Abstract

The interface between science and policy is a complex space, in theory and practice, that sees the interaction of various actors and perspectives coming together to enable policy-relevant evidence to support decision-making. Early Career Researchers (ECRs) are increasingly interested in working at the science-policy interface to support evidence-informed policy, with the number of opportunities to do so increasing at national and international levels. However, there are still many challenges limiting ECRs participation, not least how such a complex space can be accessed and navigated. While recommendations for engaging at the science-policy interface already exist, a practical ‘map’ of the science-policy interface landscape which would allow for ECR participation in evidence co-production and synthesis in science-policy is missing. With the purpose of facilitating the engagement of ECRs producing biodiversity and ecosystem services policy-relevant evidence at the interface between science and policy, the authors have co-created a ‘mind-map’—a tool to review the landscape of and leverage access to the science-policy interface. This tool was developed through reviewing published literature, collating personal experiences of the ECR authors, and validating against wider peer perspectives in an ECR workshop during the 7th Plenary of the *Intergovernmental Science-Policy Platform on Biodiversity and Ecosystem Services* (IPBES). This co-created tool sees ECR engagement in (co-)producing evidence at the science-policy interface as an interaction of three main factors: the *environment* of the ECR, which mediates their *acts of engagement* at the science-policy interface leading to *outcomes* that will ultimately have a reciprocal impact on the ECR’s environment.

**Supplementary Information:**

The online version contains supplementary material available at 10.1186/s13750-024-00334-5.

## Introduction

Globally, we are facing a set of unprecedented social-ecological crises, including dramatic losses of biodiversity, land use change, and climate change [26, 28, 53]. Addressing these challenges requires strategies informed by relevant, robust and timely social-ecological evidence [44–47, 54]. The science-policy interface is an intersectional space between science and policy at different scales is where scientists, policymakers and other actors exchange and co-produce evidence which can enrich both decision-making and/or research (van den [40, 58]). Evidence produced at the science-policy interface can be broadly defined as: “relevant information used to inform a question or decision of interest” (adopted from [49]). Researchers and practitioners in the fields of biodiversity and ecosystem services are increasingly encouraged to work at the science-policy interface as a means of supporting evidence-informed, co-created, policy-making on these crucial topics through initiatives from local, to national to global scale such as the *Intergovernmental science-policy Platform on Biodiversity and Ecosystem Services* (IPBES) [29, 46]. In the case of IPBES, and many other science-policy interfaces, a primary function is evidence synthesis or ‘assessment’ in which most up-to-date and pertinent evidence findings (in form of both academic peer-reviewed and grey literature) are normatively compiled into periodic reports for decision-makers and the general public (e.g., IPBES global, regional, thematic and methodological assessments) (Additional file 1). In civil society we expect that these evidence syntheses (i.e., assessments) will inform policy and management decisions [7] though in practice is complicated and iterative and shaped by non-linear processes of knowledge exchange.

The science-policy interface represents a theory and practice boundary between the knowledge, norms, and approaches of ‘science’ and ‘policy’. It frames evidence as a critical element in the process of developing effective policies [16]. At the interface of science and policy, the policy-making process is presented as a space for evidence gathering and interpretation to support a range of beneficial outcomes, benefitting from a solid research base which helps to ground and set the context for problem framing and policy formulation [14]. Work at the science-policy interface ideally requires a transdisciplinary^1^ approach that encourages constructive evidence exchange and co-creation between a diverse range of actors.

As noted above a range of factors impact how decision-makers use evidence including: institutional and organisational factors, characteristics of the various actors involved in evidence (co-)production, and factors affecting the direct applicability of the knowledge [48, 52, 59]. The speed of change, volume of knowledge production and differing interpretations of issues by scientists and decision-makers, common for complex and emerging topics such as climate change, can preclude relevant environmental evidence from policy arenas [42] and create bias in the issues which are engaged with. The gap between evidence and policy is further widened as scientists and policy makers can have different motivations, pressures and timescales shaping their work [31].

Work at the interface of science and policy can, therefore, be complex to navigate as it requires bringing together multiple actors with diverse knowledge and worldviews to facilitate the process of negotiation towards decision-making while navigating complex sets of priorities, jurisdictions and institutional settings [3, 37, 55]. Although bodies developing and promoting integrated approaches for connecting environmental evidence, policy and practice, such as IPBES are increasingly common and visible, the structure and operation of such efforts is complex and both the entry points and the most effective ways to contribute can be difficult to identify [3].

Authors of this article, themselves a group of ECRs, were involved in a study of ECR experience and perceptions of engaging at the science-policy interface (see also [15]). We acknowledge that the science-policy interface can seem extremely intimidating. From the perspective of an ECR, the already complex science-policy interface, with its apparently numerous but unclear points of access, can be further complicated by time and resource constraints, a lack of specialized training, limited personal networks, and a modest track record of previous personal work [15]. Working at the science-policy interface often requires engaging with unfamiliar colleagues including directly with decision-makers, representatives of important decision-making bodies, and senior scientists with many years of experience. These factors can lead to a reduced awareness of opportunities, lack of invitations to engage and lower confidence in seeking and accepting opportunities when they arise [14, 21, 33, 61].

However, ECRs can hugely benefit from working at the science-policy interface, leveraging the value of their own research, building their capacity in working with policy agendas, framing policy-relevant research questions, and identifying pathways to create social change [21]. In this article we aim to help ECRs through a co-created mind-map, which can assist ECRs in navigating and situating themselves at the science-policy interface. It builds upon pioneering work that has been published in recent years on opportunities and challenges for ECRs working at and contributing to the science-policy interface [6, 14, 15, 19, 21, 29, 51], hoping to help ECRs better understand their potential roles working at the science-policy interface and inspire and guide ECRs to get more involved. While this mind-map is targeted toward ECRs, it can also be used by boundary organizations or other organizations who have programs for ECRs contributing to the science-policy interface, to design and assess the effectiveness of their programs.

In the following sections we summarise a case for ECRs to contribute to the science-policy interface, provide a description of the process of co-creating the mind-map, and explore the ways that the mind-map can help ECRs to navigate the (co-)production of evidence and evidence-informed policy at the science-policy interface. We illustrate the mind-map using the Intergovernmental science-policy Platform for Ecosystem Services and Biodiversity (IPBES) fellows program for ECRs.

### Why should ECRs engage in work at the science-policy interface?

Knowledge transfer between evidence and policy was historically thought of as a linear and one-way process, but this view is now changing to recognize a more complex two-way relationship [11, 38, 57]. Knowledge exchange among scientists and policy-makers underpins and enables learning and evidence-informed policy-making [12, 32]. Like others, we conceptualize the interface between science and policy as a place actors can “work at” and “contribute to” [2, 9, 14, 32]. By working at the science-policy interface, ECRs active in the field of biodiversity and ecosystem services can learn how to make evidence about biodiversity loss and environmental challenges relevant to policy-makers, and ultimately issues upon which real-world action is taken. At the science-policy interface ECRs can learn to link evidence on biodiversity and ecosystem to issues at the forefront of the political agenda such as the economy, security, human health, and the Sustainable Development Goals, which have been endorsed by all countries [60]. ECRs can also learn to formulate research questions that are relevant to decision-makers and other societal actors, improving the relevance and applicability of their work [50]. Contributing to the science-policy interface can enhance more general ECR skills such as collating and communicating large volumes of often conflicting information, communicating scientific uncertainty, undertaking evidence reviews, and learning to engage with multiple actors and institutions [21, 29]. It can be crucial for forming networks with more senior scientists, decision-makers, and fellow ECRs [21].

There is much that ECRs can contribute to the co-production of policy-relevant evidence at the science-policy interface. ECRs have up-to-date understanding of research topics, many being actively involved in research themselves [5], are open to opportunities, less likely to be locked into old patterns of thinking and doing [23, 24, 41], and are likely to be willing to take an active role in informing policy-making and advocating for transformative change [46]. As the current global crises unfold, ECRs will be highly impacted by their consequences, and might feel an ever-growing motivation and responsibility to be the problem solvers [10, 30], becoming key players in intergenerational work at the science-policy interface [19, 39]. ECRs are training and working during a highly digitally connected time and can bring strong communication skills, experience in public engagement, and familiarity with emerging communication and engagement technologies which can facilitate dialogue and help engage and communicate evidence to policy-makers and wider society [8, 22, 30, 34].

## Mind-map to help ECRs navigate at situate themselves at the science-policy interface

### Developing a mind-map

The ECR-Science Policy Interface mind-map draws its overall structure from Lawson and Lawson’s [36] framework for ‘student engagement’ and was developed based on the direct experience of the authors (all ECRs working across a wide range of geographic settings) to better articulate and contextualise our own work at the science-policy interface. The mind-map was used to shape an informal ECR workshop run by the team during the stakeholder day of the 7th IPBES Plenary meeting in Paris, France (2019) from which the insights of ECRs working at the science-policy interface were then considered, helping to validate the mind map in its final form^2^. The ECR-Science Policy Interface mind-map does not seek to provide a fully comprehensive view of the experience of all ECRs but aims to give detailed qualitative insights into the perspectives of a range of ECRs actively working at the science-policy interface to understand how others can become more engaged.

The author team co-developed an original, outline version of an ECR-Science Policy Interface mind-map using the Lawson and Lawson [36] framework to make sense of their direct experiences at the science-policy interface, complemented by key literature on the topic (as summarised in Sect. "Why should ECRs engage in work at the science-policy interface?"). Lawson and Lawson [36] present their original framework (focussed on secondary and post-secondary students) as the ‘conceptual glue’ connecting the different elements of: ‘agency’ (related to prior knowledge, experience) and ‘environment/ecology’ within which individuals were operating (in relation to peers, family, and community) to the ‘organisational structures and cultures’ of their institutions. The framework provides a broad, system-oriented conceptualization that includes the psychological, sociocultural, and sociological dimensions of engagement (in this context seen as the physical, cognitive, and behavioural presence in and attentiveness to spaces and processes of learning and personal development). The author team saw commonalities with the ECR-Science Policy Interface context in this framework, finding it a useful tool for structuring and sharing experiences and undertaking reflective practice. The authors were particularly interested in understanding the nuanced relationship between opportunities, barriers to entry and benefits of working at the science-policy interface and resolved to test the utility of the mind-map as a potential tool for continuing professional learning and development for other ECRs. One opportunity to explore this was through the use of the mind-map to frame the activities conducted in a small, informal ECR workshop.

An informal 90-min-long lunchtime workshop with ECRs was designed by the author team and held during the 7th IPBES Plenary stakeholder day in May 2019 (Paris, France). The session aimed to bring together ECRs to share knowledge and experiences, but also to simply meet with other ECRs at the plenary in order to develop new professional networks (detailed methodology included in supporting information). Around thirty^3^ ECRs were recruited to the session. This was facilitated by a social media call through the authors Facebook and Twitter accounts, cascaded to professional networks, targeting ECRs attending the Plenary to participate in discussions around their engagement in SPI.

During the session the participants split into two smaller groups, to enable ease of conversation and greater opportunity for all to speak. Both groups explored two broad questions: (i) “Have you worked at the science-policy interface? If yes, in what way? If not, why not?” and (ii) What has your experience of working at the science-policy interface been like?” The draft mind-map categories were used as prompts during the discussions. Abductive thematic analysis was conducted on insights emerging in response to these questions and themes were mapped back on to the draft mind-map to consolidate the categories, identify any new categories, and verify its structure and scope.

**Fig. 1:**
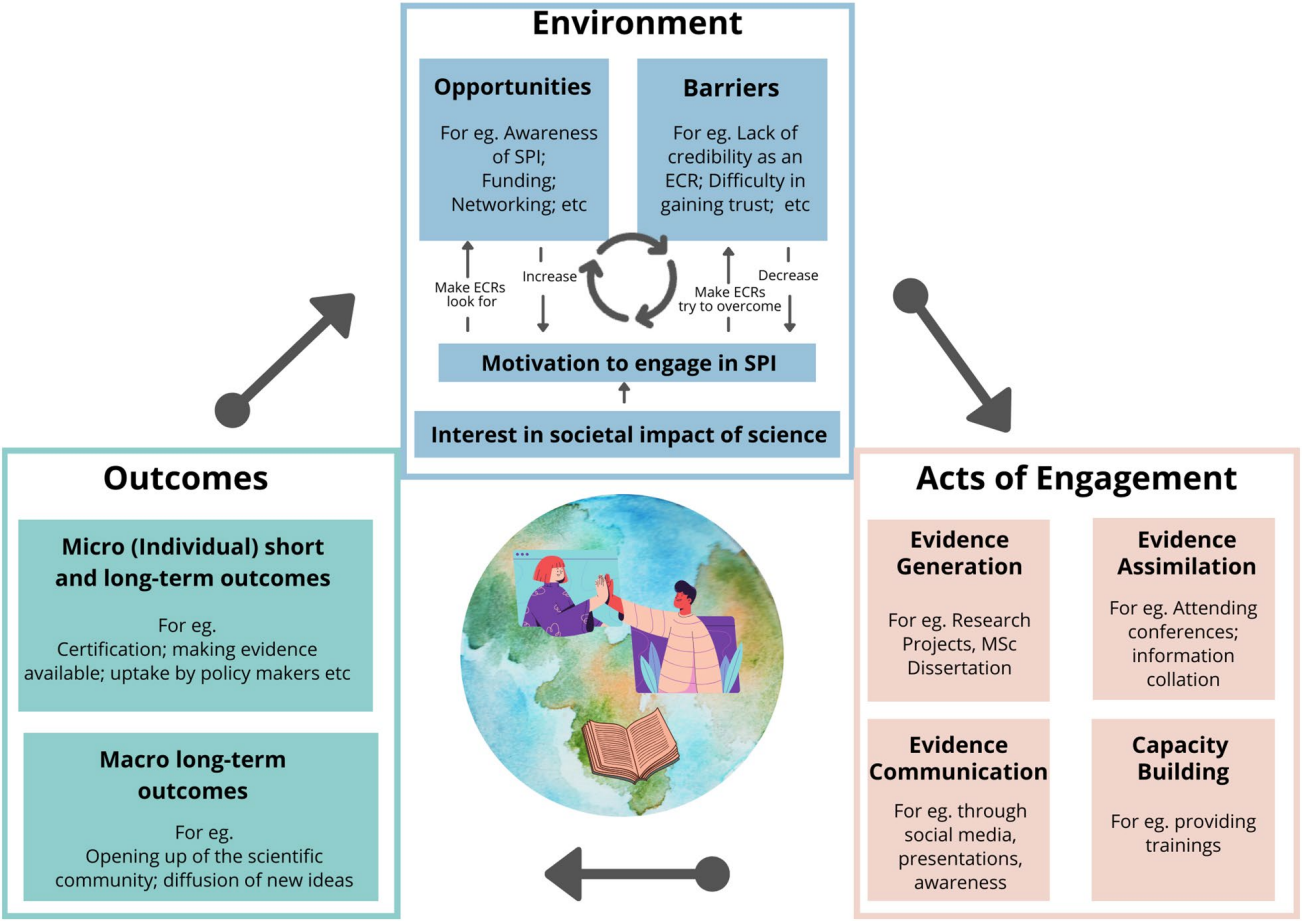
Co-created ECR-Science Policy Interface (SPI) mind-map detailing ECR engagement at the science-policy interface. ECR engagement is illustrated as a systemic process with multiple feedbacks

With reference to the final version of the co-developed mind-map the author team hypothesised that several connected factors seemed to be critical in ECR engagement at the science-policy interface: the *environment* of ECRs (in terms of motivation, opportunities and barriers), which mediates their *acts of engagement* (in relation to the science-policy interface), which then lead to various *outcomes,* ultimately feeding back into the ECR environment (Fig. 1).

### Exploring ECR experiences through the ECR-science-policy interface mind-map

The ECR-Science Policy Interface mind-map recognises that the *environment* an ECR finds themself in strongly mediates their engagement at the science-policy interface (Fig. 1). *Environment* refers to both internal (e.g. personal interests and character) and external (e.g. place of work) factors. The exercise of creating the ECR-Science Policy Interface mind-map highlighted the existence of a wide range of opportunities and barriers in the immediate *environment* that, in interaction with ECRs individual motivations, influence their engagement at the science-policy interface. These are explained further in Table 1, which provides concrete examples identified by the authors and workshop participants. “Motivation”, “Opportunities” and “Barriers” interact to determine ECRs’ level of access at the science-policy interface and the combination of these factors can vary with socio-cultural contexts. Motivated ECRs may choose to seek out, work in, or collaborate with colleagues in, environments where the contribution of evidence to policy-making is talked about and promoted, seek graduate programs or internships with a science-policy focus and join or establish networks of peers active in this space increasing awareness of opportunity and visibility to others working in the space. We note that ECRs seeking to engage in work at the science-policy interface should be mindful of, but not deterred by, others’ perceptions of their experience or credibility, which is one of the key perceived barriers. Increasing real and perceived legitimacy at the science-policy interface, through increased practical experience and personal resilience, can play a self-reinforcing role through ac*ts of engagement* at the science-policy interface, as described in the following section. The perception and reality of barriers to access and contribution can be magnified by socio-cultural and demographic factors. These factors can be real and numerous and it is important to be aware of their intersectional nature. The ‘Academic Wheel of Privilege’ developed by the UK Research Integrity Office (UKRIO) is a useful additional tool here, one of a number of approaches to recognise privilege within research contexts and highlights the importance of acknowledging the role of diverse factors including skin colour, sexuality, mental health, neurodiversity, body size, economic and social background, wealth, language, caring duties as well as a range of educational and career based factors [13]. While a broader discussion of this is beyond the scope of this paper, we recognise the need to continue to engage academic institutions and institutions and processes across the science-policy interface on the topics of privilege and inequity, as they require large-scale systemic recognition and change to address. While changes can be motivated and buoyed by individual and collective action, they should not expected to be the role and responsibility of the individual ECR. We hope that this work can add visibility and voice to that collective call.

**Table 1:** Examples of environmental factors highlighted by the authors and workshop participants

Motivation	Opportunities	Barriers
• An intrinsic interest in the societal impact of scientific evidence • A desire to build skills in science-policy • A desire to form networks with decision-makers, senior scientists and other ECR’s	• Exposure to a studying or working environment where the contribution of evidence to decision-making is talked about and promoted • Access to graduate programs or internships with a focus on working at the science-policy interface • Exposure to networking opportunities related to science-policy. Such as engagement with groups of peers (e.g. ECR networks) as a stepping stone for involvement in working at the science-policy interface	• Entry: Equity of opportunity. Socio-cultural and demograhic factors such as gender could act as barriers and / or influence perceptions of credibility and experience Contribution: • Engagement in activities related to evidence generation and knowledge production. A lack of transdisciplinarity in the academic approach to science-policy • Impact and effectiveness: Networking and visibility, interfacing with knowledge users. Perceived lack of credibility of ECRs due to limited experience and / or track record. Being early-career and / or having little experience in the field could hinder engagement with decision-makers

A*cts of engagement* refers to the activities and roles through which ECRs can engage at the science-policy interface (Fig. 1). Our work highlighted four main *‘acts’* through which ECRs can engage at the science-policy interface. These are further explained in Table 2, which provides examples identified by the authors and workshop participants. ‘Evidence Generation’, which refers to ECRs answering novel questions for policy, was identified as the main way in which ECRs are currently contributing to the science-policy interface. Here, new knowledge is usually generated. This could be done as part of the Master’s or PHD program, or through independent research projects.

**Table 2:** Examples of Acts of Engagement highlighted by the authors and workshop participants

Evidence generation	Evidence assimilation	Evidence dissemination	Capacity building
• ECRs engaged in producing reports and assessments for policy needs • ECRs engaged in answering research questions relevant for policy	• Learning about the needs of decision-makers and how to communicate with them about their research • Collating information from a variety of different sources • Evidence reviews • Integrating scientific and other kinds of evidence, including Indigenous and local knowledge	• Presenting results at events attended by decision-makers • Synthesising research outputs for members of government/parliament • Being active on social media	• Training peers or other stakeholders in science-policy, or raising awareness of science-policy issues • Increasing awareness and literacy through social media engagement

Evidence assimilation refers to the assimilation of knowledge to answer policy questions, but no new primary knowledge is created. Such activities include metanalysis, systematic literature reviews, non-systematic literature reviews, etc. Deserving of more attention is also the role played by different knowledge systems (i.e., Indigenous and local knowledge) as an element of “evidence assimilation”. There is increasing awareness on the potential of local and Indigenous knowledges informing policies, and mutual learning between Western scientific knowledge and Indigenous and local knowledge is increasingly recognized, although more efforts should be dedicated towards operationalizing this interaction (Šakić [56]). Boundary organizations, such as IPBES, engaged in this domain increasingly offer sustainability focused capacity building programs for ECRs which can provide entry points for engagement (e.g. IPBES fellowship, Evidence 4 Democracy (E4D)’s Science to Policy Accelerator, AAAS Science & Technology Policy Fellowships, Science Outside the Lab, STPI’s Policy Fellowship Program, Canadian Science Policy Fellowship by Mitacs, etc.) [1, 4, 25, 43]. These opportunities, such as the IPBES fellowship provide opportunities for ECRs to engage with diverse (i.e., western-based, Indigenous, and local) knowledge systems and knowledge holders in identifying knowledge needs of policymakers, catalyzing efforts to generate or synthesize new knowledge, and producing and delivering assessments of environmental evidence [19–21, 39]. More than 150 science-policy opportunities for ECRs have been identified across 50 countries [1], Table S1, [14, 39]. Knowledge dissemination refers to actively distributing knowledge to policy and decision-makers. We identify this as a space that ECRs are particularly well-placed to participate in due to their familiarity with different technologies and social media (see also [15]). ECRs may develop evidence dissemination skills by engaging with synthesis activities and being active in spaces outside of traditional research including social media. ECRs may also receive or offer peer training or otherwise engage in training that seeks to bring together different stakeholder groups. Some successful examples, such as the Young Ecosystem Services Specialist (YESS) group, offer spaces that ECRs can engage in capacity building activities, through peer-to-peer exchange.

The *outcomes* of these *acts of engagement* were described as ranging from “Micro” (individual and /or short-term) outcomes, to “Macro” (collective and / or long-term) outcomes (Fig. 1). These are further explained in Table 3, which provides examples identified by the authors and workshop participants. ECRs can personally seek to increase skills and knowledge relevant to the science-policy interface through their active engagement, leading to a range of both individual and collective outcomes. Training and familiarisation activities can take a range of forms, from developing skills in specific methods and approaches including evidence synthesis methods, policy writing and science communication skills; through formal workshops offered by professional or learned societies or taking place alongside or as part of conferences (e.g. ECR workshops offered as part of the Ecosystem Services Partnerships Conferences); to training to gain familiarity on topics such as the IPBES methodologies and processes (offered in open, online formats by IPBES). While engagements may be initially individually focussed and short-term, they can shape personal development trajectories over the long-term, be shared with peers and brought back to institutions, and ultimately lead to more ‘macro’ outcomes creating positive changes in the *environment* for ECRs and their colleagues.

**Table 3:** Examples of Outcomes highlighted by the authors and workshop participants

Micro (individual) outcomes	Macro outcomes
• Increase in skills and knowledge, which can take the form of contribution to evidence products such as: - scientific papers, - technical reports and - policy briefs • ECRs are also likely to help other stakeholders (students, NGOs, governments) increase their knowledge and skills by engaging in self-led or formalised training and familiarisation activities • The role of ECRs in “bringing back” knowledge to their respective institutions	• Diffusion of new ideas and ways of thinking • Impact on the way that Academia will interact with the science-policy interface in the future • Creation of a new generation of policy-savvy researchers

One of the key benefits of the ECR-Science Policy Interface mind-map is acknowledging that *environment, acts of engagement* and *outcomes* are intrinsically linked. ECRs may struggle to leverage their motivation to be involved in work at the science-policy interface in an *environment* where barriers outweigh opportunities. *Acts of engagement* can only be effectively undertaken once the *environment* permits and enables. In an ideal scenario, the *outcomes* that follow the *acts of engagement* nurture the *environment* in a positive feedback loop.

## Example: the intergovernmental science-policy platform for biodiversity and ecosystem services (IPBES) fellows programme for ECRs

In this section we illustrate how ECRs can use the mind-map to understand where to situate themselves within programs that engage at the science-policy interface. The mind-map can be used as a tool to identify entry points, skills needed, and benefits that ECRs can gain through the engagement. As noted briefly in the introduction, the Intergovernmental science-policy Platform for Biodiversity and Ecosystem Services (IPBES) is a science-policy interface boundary organization operating at the global level that assimilates and evaluates knowledge on biodiversity and ecosystem services in the form of written ‘assessments’, which are made available to member governments to use in decision-making [34]. The fellowship programme provides an opportunity for ECRs to participate in the assessment process and they are trained and supported through a variety of means such as capacity-building workshops and one-on-one mentorship with more experienced scientists [27].

ECRs might be *motivated* to participate in the fellowship programme for several reasons such as the desire to contribute to societal change, the opportunity for networking, or for academic growth [15]. However, the opportunities and barriers in the environment they work in could determine whether they have the opportunity to participate. IPBES advertises the call for the fellows programme through their website, newsletter, and social media posts [17]. This is further amplified through other mailing lists and networks. IPBES does not pay the fellows and travel support is provided only for fellows from the Global South, so universities or host institutions are expected to support fellows’ participation [27]. Finally, the fellows have to be nominated either through a country focal point or through the institution. These processes can either be opportunities or barriers for ECR engagement depending on individual contexts. As a first step, for an ECR to be aware of this opportunity to engage, the ECR needs to be in an environment where they are exposed to this information, such as through a mentor who informs them of this opportunity or through social media. Secondly, a supportive host institution is essential to cover costs and support the in-kind contribution of fellows to the IPBES process. Lastly, the process of nomination can be complicated as in some instances country focal points can be non-responsive, or would prefer to nominate candidates known to them. Having identified the barriers and opportunities using the mind-map (and tools like the UKRIO Academic Wheel of Privilege), ECRs can then recognise the parameters of these barriers and, where appropriate and proportionate, develop individual and collective strategies to address these barriers and capitalize on the opportunities. As noted in Sect. "Exploring ECR experiences through the ECR-science-policy interface mind-map", the experience of barriers is deeply nuanced and there must be an acknowledgement of the systemic nature of access to opportunity and capacity to contribute. Individual actions relevant to this example include signing up for the IPBES newsletter, which is a crucial step for knowledge about when the fellowships and other opportunities open. Building contacts within and beyond institutions is important to extend professional networks and connecting with the national focal point before-hand can be important for the nomination process. If they don’t respond, it could be important for the prospective fellow to discuss with their institution to be nominated. Increased interaction with people working at the science-policy interface can gradually reduce the barriers associated with the perceived lack of credibility and also bring ECRs in to contact with a greater diversity of actors within the system who may share or have shared similar constellations of barriers. Similarly, boundary organizations can use the mind-map to recognize what the barriers and opportunities are and make entry easier.

Assuming that the ECR has been selected for the fellowship programme, the *acts of engagement* are defined within the IPBES framework. ECRs, like the other experts in the assessment, are engaged in evidence assimilation, evidence dissemination, and capacity building [34]. The experts in the IPBES process don’t create new knowledge but collate existing information from a variety of sources. ECRs are engaged in evidence assimilation through conducting systematic and non-systematic literature reviews, integrating different forms of knowledge, and collating available information to meet the assessment needs [19]. A crucial aspect of IPBES is that the integration of multiple different forms of knowledge such as western knowledge and Indigenous and Local Knowledge through different modalities, which the fellows are extensively trained in [21].

Within IPBES, the evidence dissemination process begins after the approval of the assessment by the member governments in the Plenary. ECRs can engage in information dissemination through creative means such as creation of social media posts, writing articles in print and online media, or organizing workshops for different audiences such as policy makers, schools, or universities [18]. Opportunities for capacity building exist within the fellowship programme for the fellows themselves to help others build their capacity. Capacity building workshops and one-on-one mentorship is provided for the fellows to build their own capacity [20]. As the fellows grow in the programme, opportunities are provided to help build other capacity through the alumni networks or mentoring newer recruits. Using the mind-map to recognize the skills needed or the skills that they will gain, can help ECRs reflect on if this is the direction that they would like to grow in, if these are the skills that they would like to develop. If not, perhaps this particular opportunity is not the right one.

Fellows who have graduated from the fellowship programme have reported a range of individual outcomes such as an improvement in academic capabilities, creating an academic identity, increased knowledge of the science-policy interface, insight into international negotiation processes, and improved networks [18]. Macro level outcomes reported include: training experts to perform bigger future roles within the IPBES system, the fellows acting as force multipliers diffusing IPBES ideas and knowledge in their own environments, and intergenerational partnerships developed are crucial for planetary sustainability [39]. Mapping the micro-level and macro-level outcomes can be useful for a personal cost–benefit analysis: are the outcomes worth the investment? Will this be useful for the ECR’s career?

## Conclusion

The authors hope that this co-created mind-map provides a useful basis for guiding ECRs through some key considerations of (co-)producing policy-relevant evidence and evidence-informed policy at the science-policy interface and operates alongside existing recommendations to further encourage and enable engagement. In particular, we hope that ECRs can profit from the mind-map to:

- appreciate that working environments can present opportunities and barriers to engagement, which may need to be acknowledged and navigated to channel motivation;
- see opportunities to capitalise on activities related to evidence generation, as well as seeking less obvious but no less critical opportunities to contribute to evidence assimilation and dissemination, and capacity-building; and
- appreciate that work at the science-policy interface can help to deliver both micro- and macro-outcomes, that may help to drive positive change in the wider working environment.

We also hope that boundary organizations can use the mind-map to develop a theory of change and identify monitoring and evaluation indicators to assess the barriers to entry for and effectiveness of their programs.

This co-created mind-map is the result of ECRs discussions, leveraging their experience engaging in work at the science-policy interface. The authors have therefore presented the point of view and perceived role that ECRs play in supporting and contributing to the science-policy interface. Nonetheless, it must also be acknowledged that there is a great variety of actors driving work at and success of the science-policy interface, whose role must be further investigated in relation to ECRs and complex dynamics (e.g. influenced by context specificities). Therefore, we encourage further research in exploring how the engagement of different actors influences the science-policy interface, with the hope that this mind-map can be used as a starting point for such follow-up endeavour.

The ECR-Science Policy Interface mind-map has applicability as a tool for planning and process mapping as well as self-reflection and evaluation, as it increases the transparency of the complex factors dictating engagement and highlights the interactions between them. As well as an aid for individuals navigating the science-policy interface, we hope that it will be a useful tool for structuring and initiating discussions, experience sharing and peer-learning processes within ECR groups and in supporting discussion with colleagues and across organisations. We are keen to know if and how readers have used or interacted with the proposed mind-map and strongly encourage anyone interested in this space to get in touch to explore further engagements.

## Supplementary Information

**Additional file 1.** Methodology in (detail)

## Data Availability

Not applicable.

## Abbreviations

ECRs: Early-career researchers
IPBES: Intergovernmental Science-Policy Platform on Biodiversity and Ecosystem Services

## Footnotes

1 “Transdisciplinarity is a reflexive, integrative, method- driven scientific principle aiming at the solution or transition of societal problems and concurrently of related scientific problems by differentiating and integrating knowledge from various scientific and societal bodies of knowledge.” Lang et al. [35]

2 IPBES was established in 2012 as: “an independent intergovernmental body […] to strengthen the science-policy interface for biodiversity and ecosystem services for the conservation and sustainable use of biodiversity, long-term human well-being and sustainable development” and is a major SPI actor https://ipbes.net/

3 Participants could join or leave at any point in the session, considering the sum of full and partial attendance throughout the session this is a valid estimate. There is not a definitive total count.
