# Supplementary material for: Navigating the science policy interface: a co-created mind-map to support early career research contributions to policy-relevant evidence
Source: Environ Evid. 2024 May 24;13:15. doi: 10.1186/s13750-024-00334-5 (PMC11378860; doi:10.1186/s13750-024-00334-5)
Supplement: Supplementary file 1 — Additional file 1. Methodology in (detail) [file 13750_2024_334_MOESM1_ESM.docx]

Supporting Information

Methodology (detail)

Workshop at IPBES 7 Plenary

The author team had the opportunity to host an informal workshop as part of the IPBES 7 Plenary, with a focus on sharing insights and experiences of ECRs working at the science-policy interface. As noted in section 3.1, the team had been working on a mind-map prior to the plenary, and used this as a means to help design the session. We saw the mind-map as a potential tool for continuing professional learning and development that might be tested and shaped in to a more useful and applicable form by insights shared by the group.

This work was exempted from formal research ethics (human subject research) as it was deemed to fall under the category of: “Research involving the use of non-sensitive, completely anonymous educational tests, survey and interview procedures when the participants are not defined as "vulnerable" and participation will not induce undue psychological stress or anxiety.” (UCL Research Ethics Committee). At the point that this work was conducted, pre-registration of exempted research was not required so this study does not carry an ethics record number. Despite the lack of formal ethics registration, the team still ensured in all other respects that the work was conducted in accordance with accepted ethical standards for workshop-based research. As no personal data was collected and therefore all inputs were anonymous, the data from the workshop was not subject to specific data protection requirements, however, the team still ensured that hard copies of notes were appropriately handled and that digital versions once transposed were shared only within the team.

Participants to the workshop were recruited through a social media call, using Facebook and Twitter (with #YESS2IPBES (referring to the Young Ecosystem Services Specialists group, which many of the authors are affiliated with) and #ipbes7 hashtags), targeting any self-identifying ECRs attending the Plenary. The approximately thirty ECRs who attended (as participants could join or leave at any point in the session, there is not a definitive count) were encouraged to participate in discussions around their engagement at the science-policy interface. Participants were orally briefed on the aims of the workshop at the beginning of the session, participated voluntarily and could leave the workshop at any point.

To enable more flowing discussion and wide participation, participants were split into two smaller groups, seated at separate tables. There was almost equal gender representation in both groups and participants were from Europe, Africa, and Asia. The workshop was conducted with three to four facilitators and one note-taker from the author team in each group. Responses were collected anonymously and stored as hand-written notes by the note-taker that could not be traced back to individuals.

To establish a common understanding of the science-policy interface, we first asked each participant to share their definition of the science-policy interface. Then, we explored two different sets of questions: (i) “Have you worked at the science-policy interface? If yes, in what way? If not, why not?” and (ii) What has your experience working at the science-policy interface been like?”. If the group struggled with the broader questions, the draft mind-map categories were used more explicitly as prompts during the discussions, to explore the different experiences that the group had in their environment, what types of engagements they had been involved in, what these produced by way of outputs, and what the results and impact of these engagements had been.

Following the workshop, the handwritten notes were read, digitised, and abductively coded and cross-checked by independent pairs and trios of coders, using the code categories defined in the draft mind-map and to capture any codes and / or relationships between codes which may not have been previously captured. The team found that the insights from the group reinforced the high-level codes and code relationships captured in the mind-map, introduced some more nuanced examples (as illustrated in Tables 1-3) but did not introduce new codes or relationships.
